# Idiopathic cocoon syndrome presenting as small bowel obstruction with asymptomatic postoperative intra-abdominal collection: a case report

**DOI:** 10.1093/jscr/rjaf595

**Published:** 2025-08-06

**Authors:** Zainab Alammar, Jawad S Alnajjar, Mohammed A Almarzooq, Manal Alquaimi

**Affiliations:** Department of Surgery, King Fahd Hospital, Alahsa, Saudi Arabia; College of Medicine, King Faisal University, Alahsa, Saudi Arabia; College of Medicine, King Faisal University, Alahsa, Saudi Arabia; Department of Surgery, College of Medicine, King Faisal University, Alahsa Saudi Arabia

**Keywords:** abdominal cocoon syndrome, sclerosing encapsulating peritonitis, small intestinal obstruction, bowel obstruction, surgical emergency

## Abstract

Abdominal cocoon syndrome (ACS) is a rare cause of small bowel obstruction (SBO). Its nonspecific presentation frequently mimics more common obstructive pathologies, delaying diagnosis. A 56-year-old male presented with abdominal pain, obstipation, and vomiting. CT showed clustered ileal loops. Exploratory laparotomy revealed a thick fibrocollagenous peritoneal membrane encapsulating ischemic bowel loops. Resection of non-viable bowel and enteroenterostomy were performed. Postoperative recovery was complicated with an asymptomatic intra-abdominal collection managed with vacuum-assisted dressing. ACS poses diagnostic and therapeutic challenges. Early recognition and surgical intervention are essential to avoid complications. Clinician awareness is crucial for timely diagnosis and management.

## Introduction

Small intestinal obstruction (SBO) is a common surgical emergency [1]. Abdominal cocoon (AC), or sclerosing encapsulating peritonitis, is a rare and often underrecognized cause of SBO [2]. It is classified into idiopathic and secondary types, with the idiopathic variant typically affecting young females [3]. This report a male patient diagnosed with ACS, highlighting the atypical presentation and the diagnostic challenges and post operative course.

## Case presentation

**Figure 1:**
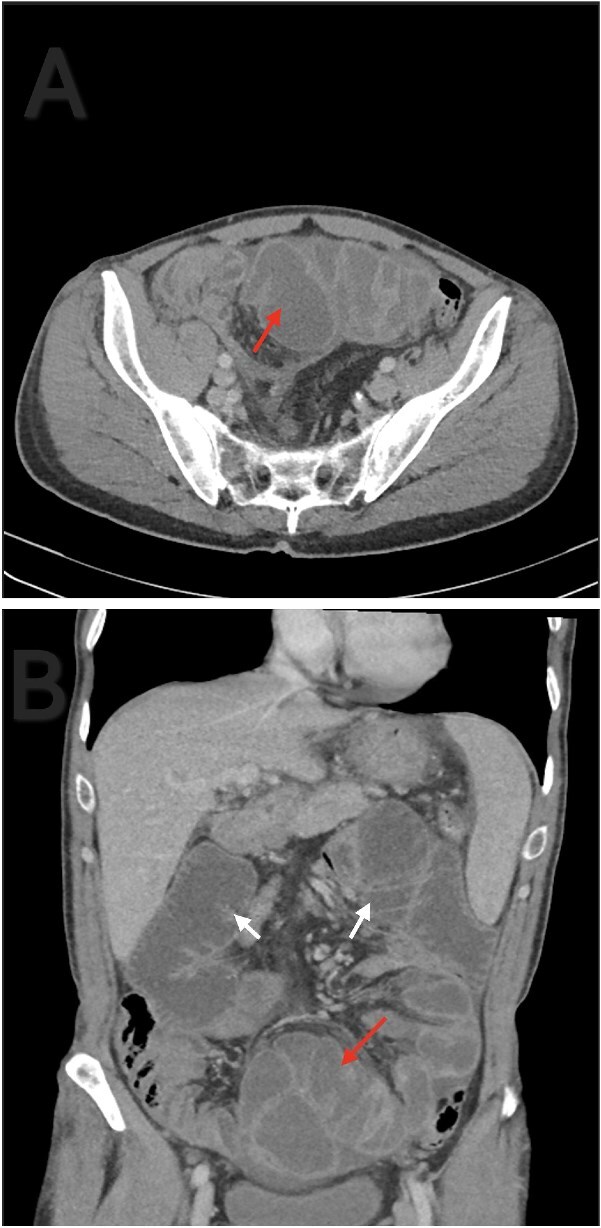
Axial (A) and coronal (B) contrast enhanced abdominal CT. Ileal loops congregated at the pelvis (red arrow). Small bowel loops dilation (white arrow).

A 56-year-old male patient presented to the emergency department with generalized abdominal pain, obstipation, and vomiting for six days. On examination, there was diffuse generalized abdominal tenderness and a palpable mass in the hypogastric region. Contrast enhanced abdominal computed tomography (CT) revealed clustered ileal loops in the pelvis with multiple transitional zones and dilated proximal small bowel loops (Fig. 1).

**Figure 2:**
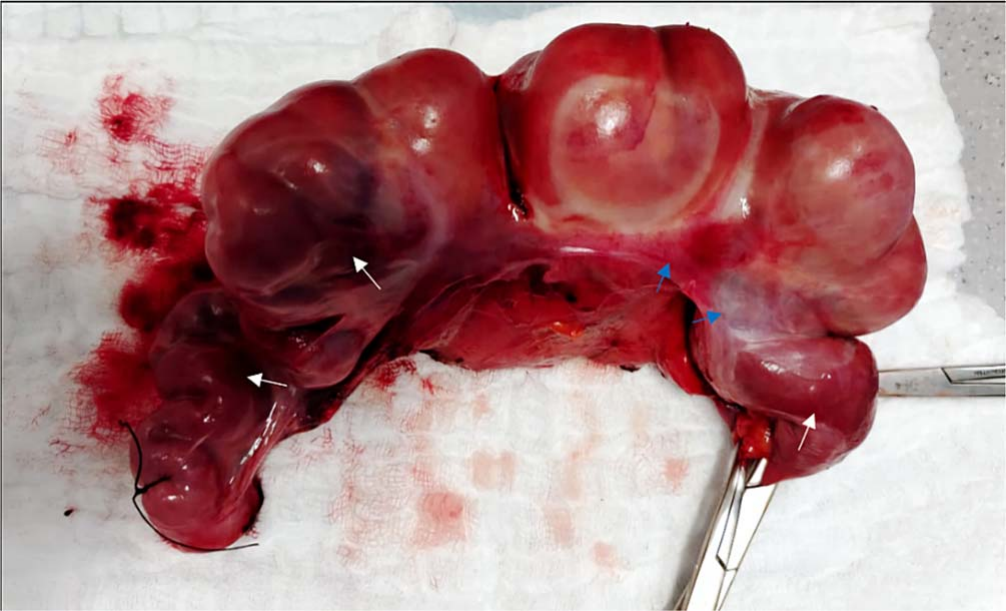
The resected segment of the terminal ilium. Ischemic bowel (white arrows). Thick peritoneum (blue arrows).

Exploratory laparotomy was performed for complete small bowel obstruction. Intraoperatively, a thick, pale peritoneum and whitish turbid fluid were found. The small bowel was encapsulated by dense adhesions. Adhesiolysis was performed, and an ischemic, dilated terminal ileum segment was resected, followed by side-to-side enteroenterostomy (Fig. 2).

**Figure 3:**
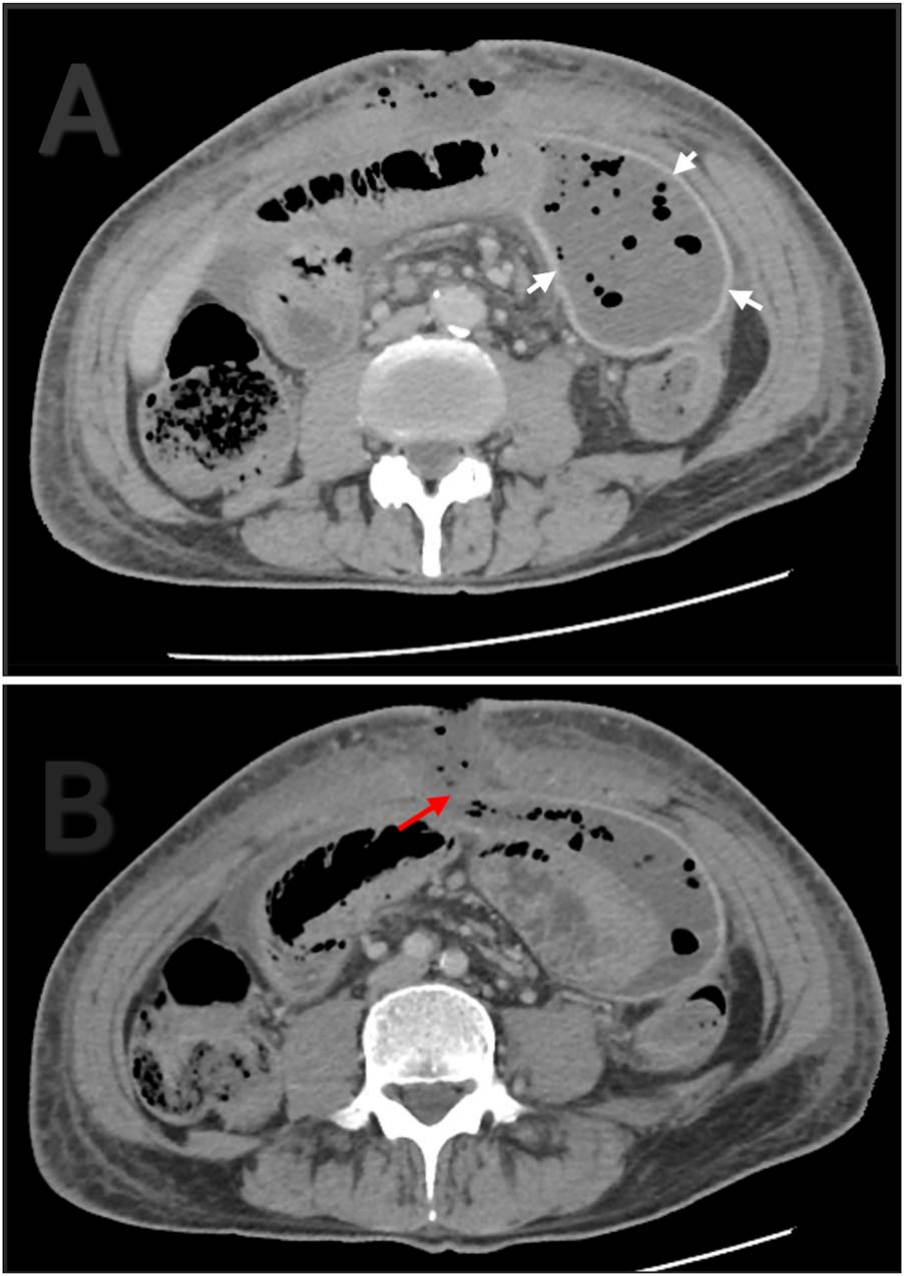
Contrast enhanced abdominal CT. (A) Thick and well localized capsule surrounding the collection (white arrows). (B) The collection is communicating with the atmosphere through a fascial dehiscence (red arrow).

The patient initially had an uneventful recovery and was discharged on postoperative day seven. A week later, he returned with asymptomatic pus discharge from the wound. CT revealed a 15 × 8 × 6 cm encapsulated intra-abdominal collection and small fascial dehiscence without signs of anastomotic leak (Fig. 3). After failed radiological drainage, a vacuum-assisted dressing was applied for 8 days. Follow-up ultrasound confirmed resolution of the collection and closure of the defect. The patient was discharged in stable condition and showed complete wound healing at follow-up.

## Discussion

Sclerosing encapsulating peritonitis (SEP) first described in 1907 as ‘peritonitis chronica fibrosa incapsulata’ by Owtschinnikow [4] and later termed abdominal cocoon syndrome (ACS) by Foo in 1978 [2]. It is a chronic inflammatory condition where the small intestines are enclosed within a dense fibrocollagenous membrane. ‘Sclerosing’ refers to collagen deposition, ‘encapsulating’ to fibrous sheath formation, and ‘peritonitis’ to the underlying inflammation [3]. The pathogenesis is linked to recurrent subclinical peritonitis, leading to fibrosis and cocoon formation [2, 5, 6]. Our patient had no risk factors, aligning with idiopathic cases reported in previous series [7].

Clinically, ACS presents variably as acute, subacute, or chronic gastrointestinal obstruction, which may be partial or complete. Common symptoms include nausea, vomiting, anorexia, weight loss, and malnutrition, though some patients remain asymptomatic. Rarely, a soft abdominal mass may be palpated [3]. In our case, the patient had a relatively short symptom duration and acute onset, which is atypical compared to reported median durations of several years [8]. ACS is classified into three types: Type 1 involves part of the small intestine, Type 2 involves the entire small intestine, and Type 3 extends to other abdominal organs [9].

CT is the imaging modality of choice, with typical findings including clustered or ‘cauliflower-like’ bowel loops and peritoneal thickening. In our patient, CT revealed clustered ileal loops and transitional zones, raising suspicion for mechanical obstruction. Histopathology, though seldom needed, can support SEP diagnosis by revealing fibroconnective tissue growth, inflammation, and dilated lymphatics. Absence of granulomas, giant cells, or birefringent material helps to rule out tuberculosis [9].

Management of abdominal cocoon syndrome depends on disease severity. Surgery is the mainstay, involving adhesiolysis and membrane excision to restore bowel function. Severe cases may require decompression or bowel resection if ischemia is present, as in our case. Conservative treatment may be adequate for mild symptoms. Incidental appendectomy is often advised [2, 9]. In this case, an ischemic ileal segment was resected followed by side-to-side anastomosis. Studies show preoperative nutritional support reduces complications, while intestinal stenting improves postoperative satisfaction [8].

## Conclusion

Abdominal Cocoon Syndrome is a rare challenging and often underdiagnosed cause of small bowel obstruction due to its nonspecific symptoms. Early recognition is essential to prevent complications such as bowel ischemia. Surgery remains the cornerstone of treatment but may be technically difficult due to extensive adhesions. More research and greater clinical awareness are needed to improve diagnosis and outcomes.
